# Neural processes in antecedent anxiety modulate risk-taking behavior

**DOI:** 10.1038/s41598-021-82229-w

**Published:** 2021-01-29

**Authors:** Kyle Nash, Josh Leota, Alex Tran

**Affiliations:** Department of Psychology, University of Alberta, Edmonton, AB T6G 2R3 Canada

**Keywords:** Human behaviour, Insula, Prefrontal cortex, Cognitive control, Decision

## Abstract

Though real-world decisions are often made in the shadow of economic uncertainties, work problems, relationship troubles, existential angst, etc., the neural processes involved in this common experience remain poorly understood. Here, we randomly assigned participants (N = 97) to either a poignant experience of forecasted economic anxiety or a no-anxiety control condition. Using electroencephalography (EEG), we then examined how source-localized, anxiety-specific neural activation modulated risky decision making and strategic behavior in the Balloon Analogue Risk Task (BART). Previous research demonstrates opposing effects of anxiety on risk-taking, leading to contrasting predictions. On the one hand, activity in the dorsomedial PFC/anterior cingulate cortex (ACC) and anterior insula, brain regions linked with anxiety and sensitivity to risk, should mediate the effect of economic anxiety on increased risk-averse decision-making. On the other hand, activation in the ventromedial PFC, a brain region important in emotion regulation and subjective valuation in decision-making, should mediate the effect of economic anxiety on increased risky decision-making. Results revealed evidence related to both predictions. Additionally, anxiety-specific activation in the dmPFC/ACC and the anterior insula were associated with disrupted learning across the task. These results shed light on the neurobiology of antecedent anxiety and risk-taking and provide potential insight into understanding how real-world anxieties can impact decision-making processes.

## Introduction

Decisions are heavily influenced by integral emotions, or emotions elicited directly by behavioral options in a decision or task at hand^1–3^. For example, rewarding and risky options elicit anticipatory emotions that guide towards or away certain choices^4^. Neural processes in integral emotions are well demonstrated and inform understanding of various behavioral and clinical issues^5,6^. Incidental emotions, or emotions unrelated to the decision or task at hand, also impact decisions^7–9^. For example, incidental anxiety to the threat of shock modulates activation in brain regions involved in decision-making and this activation modulates risk-taking^10^. A better understanding of neural processes in incidental emotion and decision-making may shed light on irrational choices and problematic behaviors^11^. However, research on neural processes in incidental anxiety is only in incipient stages.

Real-world decisions, from the mundane to the momentous, are often made in the shadow of anxiety. Economic uncertainties, looming deadlines, relationship dilemmas, existential threats, etc., are all common anxiety-provoking events. Prior neuroeconomic research has exclusively examined *coincidental* anxiety manipulations—i.e., manipulations *during* the risk-taking task in a within-subjects design. This approach has provided key insights into the neurobiology of decision-making processes. However, research has yet to demonstrate the neurobiology of *antecedent* anxiety on subsequent decision-making—i.e., anxiety manipulated *before* a decision-making task. Whereas coincidental anxiety manipulations presume that anxiety can be flipped on and off from trial to trial, anxious worry over important events tends to persist and is difficult to regulate^12,13^. Research on the neural processes in antecedent anxiety could shed light on how and why real-world anxious events modulate downstream decision-making^14^.

Anxiety is a distinct affective state of uncomfortable arousal and heightened vigilance caused by conflict, uncertainty, and novelty^15^. Risk-aversion may be considered a central consequence of anxiety^16^. Indeed, state and trait anxiety have been linked to increased sensitivity to risk^9,17^. However, incidental anxiety is sometimes associated with increased risk-taking. For example, incidental anxiety has been linked to problem gambling^18^, drug-seeking^19^, and unsafe sex-practices^20^. Anxiety manipulations can cause increased risk-taking^21,22^. Anxiety-inducing events also heighten extracellular dopamine in the prefrontal cortex (PFC), which increases drug cravings in individuals with substance use disorders^23^. Anxiety may increase risk-taking because it disrupts cognitive control^13^ or co-occurs with stress-induced hypersensitivity to reward^23^. Despite the real-world significance, the neurobiology of these diverging reactions remains unexplored.

Notably, there is significant overlap between cortical areas involved in decision-making and anxiety, including the dorsomedial PFC (dmPFC), the anterior cingulate cortex (ACC), the ventromedial PFC (vmPFC), and the insula^24–28^. These cortical regions reflect likely regions of interaction between emotion and decision making. To our knowledge, no study has examined if neural processes specific to antecedent anxiety modulate downstream risky decision-making. Here, we manipulated antecedent anxiety in a between-groups design and measured state brain activity using EEG and source localization (sLORETA^29^). To manipulate real-world anxiety, we randomly assigned participants to either a poignant experience of economic anxiety or a no-anxiety control condition. We then examined risk-taking behavior using the Balloon Analogue Risk Task (BART^30^).

Prior research indicates that slow-wave oscillations, including delta, theta and alpha1 bands, are inversely related to cortical activation and faster-wave oscillations, including alpha2, beta 1–3, and gamma bands, are positively related to cortical activation^31–34^. Based on this, we interpreted increases in slow-wave oscillations as decreases in cortical activation and increases in faster-wave oscillations as increases in cortical activation. Research on source localized EEG oscillations has found that anxiety involves the same brain areas implicated in other imaging research^14,24,35,36^. For example, an anxious-worry manipulation that targets personal obsessions amongst OCD patients caused increased upper beta power, or increased cortical activation, in the ACC and vmPFC^37^. Distressed participants, compared to participants that completed a relaxation task, demonstrated decreased alpha, or increased cortical activation, in the dACC^38^. Anxiety induced through hypnosis, compared to relaxation, increased upper beta power in the right PFC, particularly in more ventral regions^39^. In the melancholic, anxiety, and not depression symptoms, correlated with upper beta in the right vmPFC and dorsal PFC^40^. Finally, trait anxiousness has been associated with decreased theta power, or increased cortical activation, in the anterior insula^41^.

These considerations and the bidirectional effect of anxiety on risk-taking led to contrasting predictions. Given that anxiety can promote risk-aversion through heightened sensitivity to risk, we expected that economic anxiety would cause increased activity in the dmPFC/ACC and anterior insula, brain regions tightly linked with anxious worry and sensitivity to risk^35,36^, and this anxiety-specific activation should predict risk-averse decisions. That is, dmPFC/ACC and anterior insula activation would mediate an indirect effect of economic anxiety on more risk-averse decision-making. Alternatively, given that anxiety can promote risk-taking, either through disrupted control and/or heightened sensitivity to reward, we expected that economic anxiety would cause increased activation in the vmPFC, a brain region important in emotion regulation and subjective valuation in decision-making^14^, and this anxiety-specific activation should predict more risky decisions. That is, vmPFC activation would mediate an indirect effect of economic anxiety on more risky decision-making.

Finally, we computed two additional behavioral variables, behavioral control and learning, to examine if the effect of anxiety-specific activation was specific to risky decision-making or if it impacted other behavioral outcomes. We wished to disentangle behavioral control and learning from risk-taking given that most people display a general tendency towards risk-aversion^42,43^. Thus, increased risk-taking scores might occur due to increased variability in responding or learning a more optimal, less risk-averse, response strategy.

## Results

### Economic anxiety increases self-reported anxiety

We first examined direct effects of the manipulation on affect and behavior. We computed a Felt Anxiety composite, comprised of self-reported levels of *anxious*, *uncertain*, and *frustrated* during the manipulation. Note that these self-report analyses constitute a partial re-analysis of data published in Nash et al^44^. In a one-way ANOVA, with condition as the between-subjects variable and Felt Anxiety as the dependent variable, results showed that participants in the Economic Anxiety condition reported significantly higher levels of Felt Anxiety (*M* = 3.788, *SD* = 0.876) than participants in the No-Anxiety Control condition (*M* = 2.318, *SD* = 0.928), *F*(1, 95) = 63.744, *p* < 0.0001, η^2^_*p*_ = 0.402. This confirms that our manipulation caused self-reported anxiety. To determine if the effect is specific to felt anxiety, we computed two other composites, a Positive Affect score (items: Good, Happy, Smart, Successful, Likeable, and Meaningful) and a Negative Affect score (items: Confused, Empty, Ashamed, Insecure, Lonely, Stupid, Out of Control, and Angry). In one-way ANOVA analyses, we found that the Economic Anxiety condition caused increased Negative Affect (*M* = 2.560, *SD* = 0.787), compared to the No-Anxiety Control condition (*M* = 1.884, *SD* = 0.766), *F*(1, 95) = 17.943, *p* < 0.0001, η^2^_*p*_ = 0.159. Similarly, a second one-way ANOVA revealed that the Economic Anxiety condition caused decreased Positive Affect (*M* = 2.324, *SD* = 0.546), compared to the No- Anxiety Control condition (*M* = 3.263, *SD* = 0.655), *F*(1, 95) = 59.173, *p* < 0.0001, η^2^_*p*_ = 0.384. However, if Felt Anxiety is entered as a covariate, the effect of condition on Negative Affect is eliminated, *F*(1, 95) = 1.973, *p* = 0.163, though the effect on Positive Affect remains significant, *F*(1, 95) = 25.103, *p* < 0.0001. Finally, if either score, Negative Affect or Positive Affect, is entered as a covariate, the condition effect on Felt Anxiety remained significant, *both p’s* < 0.0001. This demonstrates that the effect of condition specifically increased Felt Anxiety and not a more general negative affect.

### Economic anxiety modulates dmPFC/dACC, vmPFC, and insula activation

**Figure 1:**
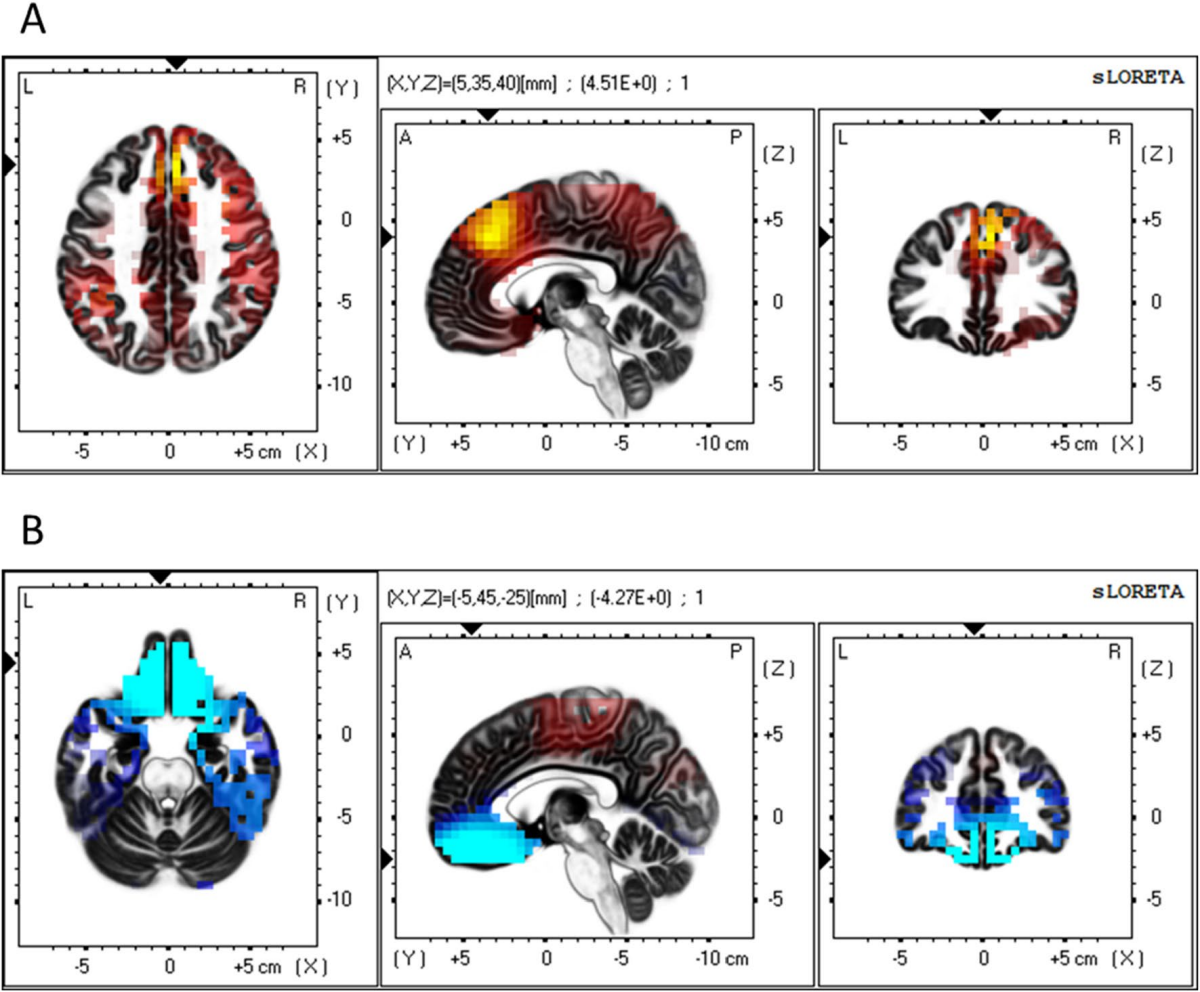
Source localization (sLORETA) results showing voxels with significantly higher activation in the Economic Anxiety condition than in the No-Anxiety Control condition. (**A**) dmPFC/dACC activation. Significant voxels in yellow, critical *t*-value > 3.835). Arrows at peak voxel, MNI coordinates = 5, 35, 40, *t*(95) = 4.510. (**B**) vmPFC activation. Significant voxels in light blue, critical *t*-value > 3.776). Arrows at peak voxel, MNI coordinates = 5, 45, − 25, *t*(95) = − 4.270.

In whole-brain, voxel-by-voxel independent groups *t*-tests of the sLORETA images for each EEG frequency band (corrected *p* = 0.05), controlling for baseline eyes-closed sLORETA images, results revealed that the Economic Anxiety manipulation caused increased beta2 frequency activation (corrected *t*-value threshold = 3.835) in a cluster of 48 voxels in the right dmPFC and dACC, peak voxel: MNI coordinates X = 5 Y = 35 Z = 40, *t*(96) = 4.51, *p* = 0.000018. The cluster included voxels in the medial frontal gyrus, the superior frontal gyrus, and the cingulate gyrus, in Brodmann Areas 6, 8, 9, and 32 (see Fig. 1A). Because activity in the beta2 frequency band is associated with increased cortical activation, these results demonstrate that the Economic Anxiety condition causes increased dmPFC/dACC activation.

Further, results revealed that the Economic Anxiety condition, compared to the No-Anxiety Control condition, caused decreased delta frequency activation (corrected *t*-value threshold = 3.776) in a cluster of 182 voxels in the vmPFC, OFC, and subgenual ACC, peak voxel: MNI coordinates X = 5 Y = 45 Z = − 25, *t*(96) = 4.27, *p* = 0.000046. The cluster included voxels primarily in the orbital gyrus, rectal gyrus, and medial frontal gyrus, in Brodmann Areas 11, 47, and 25 (see Fig. 1B). Because activity in the delta frequency is associated with decreased cortical activation, these results demonstrate that the Economic Anxiety condition causes increased vmPFC activation. Activation in all other frequencies did not cross the critical probability threshold.

In region of interest analyses focused on anterior insula activation, results revealed that that the Economic Anxiety condition, compared to the No-Anxiety Control condition, caused increased scores on the Right Insula Activation variable, *F*(1, 95) = 6.647, Bonferroni corrected *p* = 0.046 All other insula variables were non-significant (*ps* > 0.3). This suggests that the Economic Anxiety condition, compared to the No Anxiety Control condition, caused increased activation in a brain region reliably related to anxious worry and risk-aversion, the right anterior insula.

### Economic anxiety modulates risky decision-making and behavioral control

We next examined the effect of our manipulation on behavior in the BART. As indicated in the methods, we calculated two variables:

(i) *Average Adjusted Pumps in the BART (AvAdj*) Participants pumped 20 balloons in a computer game. Each pump inflated the balloon and earned points but also increase the risk of popping. Popped balloons earned no points and participants could stop anytime to collect the points earned on that trial. Our primary measure of risk-taking was the average number of pumps on trials without explosions.
(ii) *Coefficient of Variability (COV)* We computed a second measure of strategic behavior and risk-taking, the coefficient of variability (COV). COV is computed as the standard deviation across trials divided by average pumps across trials. Lower scores reflect more strategic behavior and higher scores reflect more disinhibited decision-making.

We conducted separate one way ANOVAs, again with condition as the between-subjects variable, for the two BART variables. Results revealed that participants in the Economic Anxiety condition demonstrated significantly lower levels of COV (*M* = 0.422, *SD* = 0.104), compared to participants in the No-Anxiety control condition (*M* = 0.509, *SD* = 0.156), *F*(1, 95) = 6.471, *p* = 0.013, η^2^_*p*_ = 0.064. There was no direct effect of condition on AvAdj (Economic Anxiety *M* = 9.196, *SD* = 3.508; No Anxiety Control *M* = 9.483, *SD* = 4.090, *F*(1, 95) = 0.139). However, with standard deviation across trials entered as the covariate, participants in the Economic Anxiety condition demonstrated marginally higher levels of AvAdj (*M* = 0.300, *SD* = 1.477), compared to participants in the No-Anxiety control condition (*M* = − 0.393, *SD* = 1.990), *F*(1, 95) = 3.875, *p* = 0.052, η^2^_*p*_ = 0.039. This suggests that antecedent anxiety causes increased cognitive control in the BART but has no direct impact on risky decision-making. However, if behavioral variability is controlled for in the analyses, then antecedent anxiety causes marginally higher levels of risk-taking across all trial types. More broadly, this supports the importance of considering both behavioral variability and risk-taking in this task.

### Anxiety-specific activation modulates risky decision-making and behavioral control

Anxiety-specific activation was computed by averaging the frequency-specific activation in all voxels within a radius of 10 mm around the peak voxel of each cluster or region of interest identified above, i.e., beta2 activity in the dmPFC/dACC, delta activity in the vmPFC, and activation-related frequency bands in the right anterior insula.

Bootstrapped mediational analyses (PROCESS model 4, 5000 bootstrap samples for 95% confidence intervals, see^45^) were conducted in which the predictor (X) was the condition variable, the mediator (M) was delta activation in the vmPFC, the dependent variable (Y) was average adjusted pumps (AvAdj). These analyses revealed that the Economic Anxiety manipulation had an indirect effect on AvAdj in the BART through vmPFC activation. Results showed that, as already shown above, the Economic Anxiety condition was associated with decreased delta activation in the vmPFC, and delta activation in the vmPFC was associated with reduced AvAdj, *indirect effect coefficient* = 0.4367, *SE* = 0.2788, 95% *CI* [0.0012, 1.0949] (CIs do not contain zero). Recall that delta activity is inversely related to cortical activity. Together, then, these results suggest that antecedent vmPFC activation specific to anxiety increased risk-taking in the BART.

Bootstrapped mediational analyses were also conducted with Right Insula Activation as the mediator. Results showed that the Economic Anxiety condition was associated with increased activation in the right insula and this activation was associated with reduced AvAdj, *indirect effect coefficient* = 0.4367, *SE* = 0.2788, 95% *CI* [0.0012, 1.0949] (CIs do not contain zero). These results suggest that antecedent right anterior insula activation specific to anxiety decreased risk-taking in the BART. Analyses with dmPFC/dACC beta2 activation found no significant mediational links with adjusted average pumps.

The Economic Anxiety manipulation also had an indirect effect on COV, again through vmPFC activation. Economic Anxiety was associated with decreased delta activation in the vmPFC, and delta activation in the vmPFC was associated with reduced COV, *indirect effect coefficient* = 0.0218, *SE* = 0.0091, 95% *CI* [0.0061, 0.0415]. These results similarly suggest that antecedent vmPFC activation caused by economic anxiety decreased cognitive control in the BART. Analyses with dmPFC/dACC and right anterior insula activation found no significant mediational or correlational links with COV.

#### Combined model

**Figure 2:**
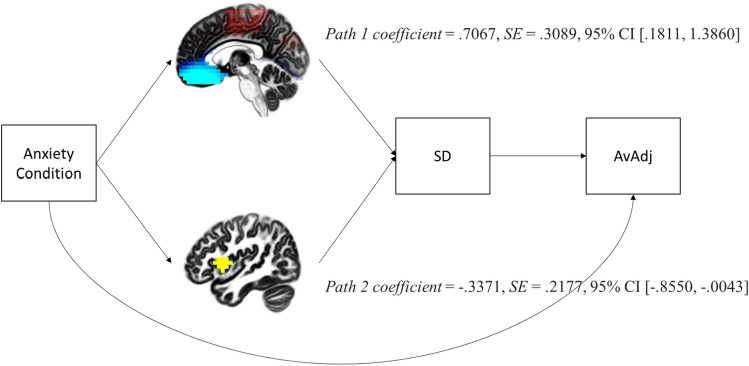
Bootstrapped parallel mediational model (PROCESS model 80; 5000 bootstrap samples for 95% confidence intervals). Path 1 is via delta band activation in the vmPFC (M1, peak voxel MNI coordinates = 5, 45, − 25). Path 2 is via right anterior insula activation (M2, peak voxel MNI coordinates = 45, 10, 5). Indirect effect coefficients confidence intervals for both paths do not contain zero, indicating the anxiety condition (i) causes decreased delta band activation in the vmPFC and this activation leads to decreased behavioral control and increased risk-taking, and (ii) causes increased right insula activation and this activation leads to increased behavioral control and reduced risk-taking.

The above suggests that antecedent anxiety has different effects on risky decision-making through different brain areas—the vmPFC and the right anterior insula. To examine this possibility of parallel processes, we tested a bootstrapped mediational model (PROCESS model 80; 5000 bootstrap samples for 95% confidence intervals, see Hayes, 2017) in which the vmPFC (M1) and the right anterior insula (M2) are entered as parallel mediators that can influence a third mediator (M3), behavioral variability, and behavioral variability influences risky decision-making (see Fig. 2). We used standard deviation (SD) across trials rather than COV given that COV is computed using average pumps, so a relationship between these two variables may not be genuine. Results demonstrated that each full indirect path was significant (X → M1 or M2 → M3 → Y). First, Economic Anxiety had a positive effect on AvAdj through increased vmPFC activation (i.e., decreased delta) and increased behavioral variability, *indirect effect coefficient* = 0.7067, *SE* = 0.3089, 95% *CI* [0.1811, 1.3860]. This finding suggests that increased vmPFC activation led to increased risk-taking through a decrease in behavioral control. Second, Economic Anxiety had a negative effect on AvAdj through increased right anterior insula activation and decreased behavioral variability, *indirect effect coefficient* = − 0.3371, *SE* = 0.2177, 95% *CI* [− 0.8550, − 0.0043]. This finding suggests that increased right anterior insula activation led to decreased risk-taking through an increase in behavioral control.

#### Learning

Finally, we examined if anxiety-specific activation had an impact on learning in the BART, i.e., changes in behavioral responding that reflect a shift towards more optimal decision-making. As described in the Methods, we computed a behavioral Learning variable. A higher score on this Learning score indicates that participants learned to approach optimal risk-taking.

**Figure 3:**
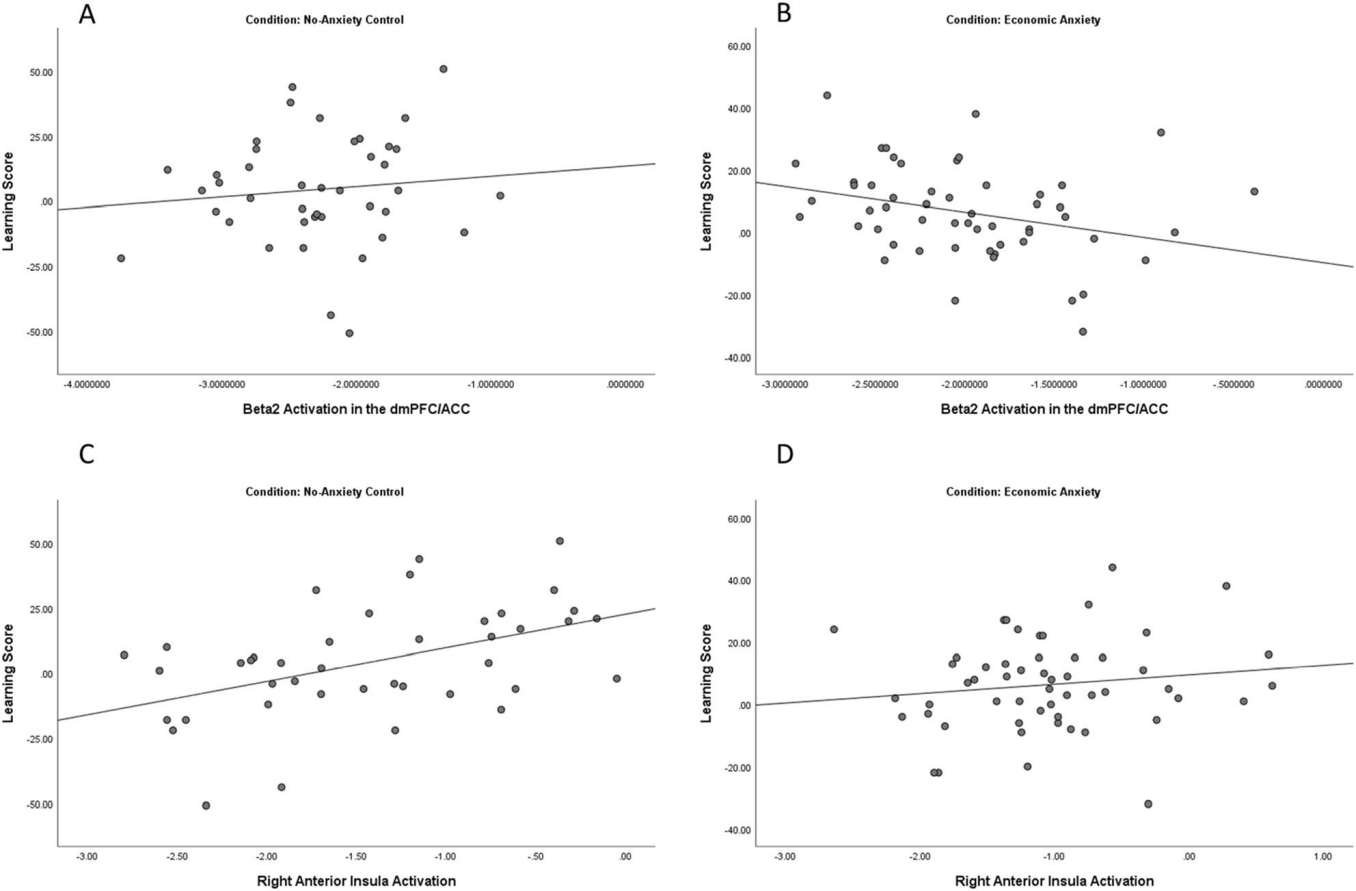
Scatterplots showing correlations between anxiety-specific activation and the learning score in the different conditions. Beta2 activation in the dmPFC/ACC in (**A**). the No-Anxiety Control condition, *r* = 0.111, *p* = 0.489, and (**B**) the Economic Anxiety Condition, *r* = − 0.305, *p* = 0.025 Right Anterior Insula Activation in (**C**) the No-Anxiety Control condition, *r* = 0.474, *p* = 0.002, and (**D**) the Economic Anxiety Condition, *r* = 0.142, *p* = 0.302.

Pearson correlations in each condition revealed that beta2 activation in the dmPFC/ACC was negatively associated with the Learning score in the Economic Anxiety condition, *r* = − 0.305, *p* = 0.025, but not in the No-Anxiety control condition, *r* = 0.111, *p* = 0.489 (see Fig. 3A,B). Given that the beta2 frequency band is positively associated with cortical activation, this suggests that anxiety-specific activation in the dmPFC/ACC was associated with initially more risk-seeking that switches to a less optimal risk-aversion. This further explains why dmPFC/ACC activation was not related to risk-taking overall. In contrast to the dmPFC/ACC finding, Right Insula Activation was associated with the Learning score in the No-Anxiety Control condition, *r* = 0.474, *p* = 0.002, but not in the Economic Anxiety condition, *r* = 0.142, *p* = 0.302 (see Fig. 3C,D). This indicates that in the control condition, anterior insula activation predicts relatively higher levels of risk-aversion that switches to more optimal risk-taking. However, in the anxiety condition, anxiety-specific activation in the anterior insula no longer predicts an increase in optimal risk-taking. Finally, delta activation in the vmPFC did not correlate with the Learning score in either condition.

## Discussion

Rarely, in everyday life, must we make a series of decisions as anxious events flit in and out of awareness. Rather, we often face looming anxieties that spill over into the decisions we make. Here, we experimentally induced this real-world experience, in which we examined how antecedent anxiety and the accompanying neural processes modulated decision-making in a risk-taking task. Based on past research demonstrating that anxiety can have diverging effects on risk-taking, we formulated contrasting predictions. An anxious experience should modulate dmPFC/dACC and anterior insula activity, brain regions tightly linked with anxious worry^35,36,46^, and this anxiety-specific activation should predict more risk-averse decisions in the BART. Alternatively, anxiety should modulate activation in the vmPFC, a brain region important in emotion regulation and decision-making^1,14^ and this anxiety-specific activation should then predict more risk-seeking decisions in the BART, through disrupted cognitive control or heightened sensitivity to reward.

We found evidence related to both predictions. On the one hand, right anterior insula activation specific to antecedent anxiety predicted decreased risk-taking. This finding is consistent with considerable research on the neural mechanisms of risk^26,47^ and the limited prior research on incidental anxiety and decision-making. For example, the threat of shock during a decision-making task increased the anterior insula’s coding of negative evaluations and this activation predicted increased rejection rate of risky lottery decisions^10,48,49^. For the first time, we extend these prior results to antecedent anxiety. The experience of economic anxiety is a poignant and difficult to regulate event^50^. Presumably, right anterior insula activation caused by the economic anxiety manipulation sustained a more cautious approach to negative outcomes that trickled-down to risk-averse decision-making.

On the other hand, vmPFC activation specific to antecedent anxiety predicted increased risk-taking. This finding is partially consistent with prior research on incidental anxiety, in which vmPFC activation was positively related to increased risk-taking^10^. However, the threat of shock decreased rather than increased vmPFC activation in this prior research. In the current study, anxiety-specific activation in the vmPFC may reflect a similar process, i.e., a change in subjective valuation, but to different types of anxious events. Whereas the discrete experience of the threat of shock temporarily reduces vmPFC activation and mutes neural valuation of positive outcomes, the increase in vmPFC activation demonstrated here may reflect an increase in neural valuation of positive outcomes and/or a shift toward a more reward-focused orientation. This is consistent with research in which anxiogenic experiences cause increased dopamine release in the PFC and this heightens sensitivity to reward^23^.

Alternatively, the vmPFC is critical in emotion regulation, particularly in terms of providing top-down control of amygdala activation to negative stimuli and affect^51–54^. Notably, research shows that people with poor regulation ability (i.e., the depressed) show increased vmPFC activation to negative affect but positive rather than negative connectivity with the amygdala^55^. The anxiety-linked vmPFC activation demonstrated here could reflect efforts to regulate anxious worry in the economic condition, which in turn lead to disruption of emotion regulation in the risk-taking task. Note that these two accounts are not exclusive, as heightened reward sensitivity can hinder self-regulation or cognitive control^56^.

Broadly consistent with the integral role of regulatory processes, we found that a behavioral measure of cognitive control was central to understanding the effects of antecedent anxiety on risky decision-making. Increased vmPFC activation mediated a decrease in an established measure of behavioral control in the BART^57,58^. Moreover, in a combined model, the decrease in behavioral control itself mediated increased risk-taking. This suggests that vmPFC activation specific to antecedent anxiety led to disrupted cognitive control, which then led to increased risk-taking. On the other hand, in the same model, increased right anterior insula activation mediated an increase in behavioral control, and this increase in behavioral control mediated decreased risk-taking. This suggests that anterior insula activation specific to antecedent anxiety led to increased cognitive control, which then led to more cautious decision-making.

Finally, we found that anxiety-specific activation had an impact on learning in the BART. First, anxiety-specific dmPFC/ACC activation was associated with decreased learning. Notably, dmPFC/ACC activation was related to initially risky decision-making that gives way to a more cautious approach, a pattern of responding that directly contrasts with learning a more optimal rate of responding. These findings mirror research in which clinical levels of anxiety disrupts extinction learning and retention^59^. Second, learning in the No-Anxiety Control condition was associated with anterior insula activation. However, anterior insula activation caused by the Economic Anxiety condition disrupts this association with learning. Because anterior insula activation is associated with coding of negative outcomes, this suggests that anxiety-specific activation in the anterior insula disrupts integration of negative outcomes into learning-related processes.

We note certain limitations and opportunities for future research. First, this study is novel as a demonstration that anxiety-specific activation due to an uncomfortable but unfortunately common experience influences subsequent decision-making. However, because we prioritized examining this somewhat distal link and avoided the issue in which participants might connect the anxiety manipulation to the risk-taking task, it is unclear how antecedent anxiety-specific activation influences later neural processes during decision-making. Presumably, such neural processes will reflect those demonstrated in prior research, i.e., online activation in the vmPFC, ventral striatum, and insula should modulate risky decision-making^25–28^. Future research could further establish the full downstream effects on neural processes by also measuring activation during risk-taking. Second, our results suggest that individual differences in reactions to anxiety may moderate how this uncomfortable affect influences decisions. That is, certain people may be more or less inclined to react to anxious experiences in ways that activate the anterior insula versus the vmPFC. Individual differences in motivational orientation appear clearly relevant^60^. For example, prior research shows that harm avoidance and neuroticism are associated with anterior insula activation during risk-taking^61^. Finally, another key region in both anxiety and decision-making is the amygdala. Our current EEG and sLORETA methodology did not allow us to image the amygdala response to the economic anxiety manipulation. Future research could examine the impact of anxiety-specific amygdala activation on later risk-taking behavior. Further, such research could examine vmPFC-amygdala connectivity to shed light on the role of top-down regulation of antecedent emotion in decision-making.

Overall, the current findings hold promise for helping explain certain irrational behaviors or problematic choices, with particular relevance for economic anxiety. For example, the 2008–09 global recession had profound emotional and social consequences^62–65^. Widespread economic anxiety looms again at the time of writing in the context of the COVID-19 pandemic. The current results represent how distal, antecedent anxieties with limited functional connection to later decision-making may subsequently incline one towards risk-aversion or risk-seeking. These anxiety-driven processes could potentially extend to other decisions related to trust, greed, dishonesty, altruism, and so on. Further, these findings may hold implications for anxiety disorders and anxiety-related traits and how these individual differences might influence decision-making. Rather than presuming that people prone to anxiety have behavioral issues due to constantly-engaged anxiety, these results suggest that disrupted behavior may be a result of antecedent anxious experiences, particularly for strategic or controlled behavior. Indeed, trait anxiety is associated with limited control capacity^13^. To improve decision-making outcomes for anxiety-related clinical populations characterized by disrupted or disinhibited behavioral profiles, one might directly target cognitive control abilities rather than reducing anxious emotions.

## Methods

### Participants

Ethical approval for this study was provided by the University of Alberta Human Research Ethics Board (Protocol 00084513), and all research was performed in accordance with relevant guidelines and regulations. The experiment used a between-subjects design with random assignment into two experimental conditions (Anxiety vs. No-Anxiety Control). Participants (*N* = 106; modal age = 19; females = 61) with normal or corrected-to-normal vision and were recruited from a first-year psychology class and earned class credit. Based on pilot data indicating that the current manipulation had a medium to large effect size on anxiety, (Cohen’s *d* = 0.65), we aimed to include 50 individuals per condition and stopped collection at the end of the fall term (power analyses in G*Power: difference between two independent groups, *expected* effect size *d* = 0.65, *alpha* = 0.05, *power* = 0.80, and *number of groups* = 2, *output* total *sample size* = 60). A total of nine participants were excluded due to noisy or missing EEG data, leaving 97 participants for analyses. One outlier was detected on one dependent measure only (activation in the dmPFC/ACC, detected by inspection of boxplots). Excluding this participant does not change the following results, and was left in for completeness (all analyses available on request).

### Experimental design

**Figure 4:**
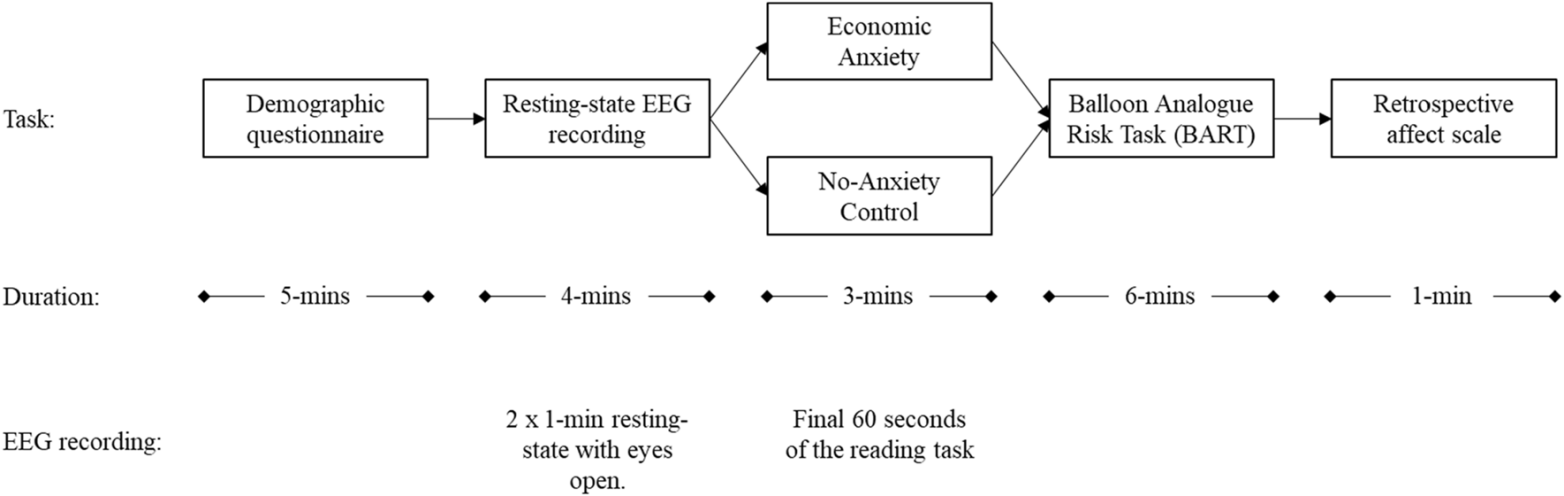
Flow chart of experimental procedure, duration of each task, and position of EEG recordings.

Participants were welcomed and first completed an electronic informed consent. They were then fitted with a 64-channel EEG headset (Brain Products) and seated at a computer station in an electrically- and sound-shielded room. Participants then answered demographic questions and several personality questionnaires as part of a broader research initiative on individual differences in the neuroscience of self-regulation (total 91 items, all materials and data available upon request, see also supplementary material 1). Next, participants were instructed to minimize movements and completed a resting-state EEG recording, alternating 1-min eyes open and eyes closed sections for a total of four minutes. Participants were then randomly assigned to either the *Anxiety* condition or the *No-Anxiety Control* condition. Again, as part of the broader research initiative, participants then completed a passive auditory startle task and a color-naming Stroop task (oddball data, see^44^; Stroop data, manuscript in prep., all data available upon request). After, participants completed the Balloon Analogue Risk Task, the behavioral measure of risk-taking. Participants then rated the degree to which the economic anxiety manipulation made them feel a range of different positive and negative emotions, including anxiety and uncertainty (1 = Strongly Disagree, 5 = Strongly Agree), and then completed a measure of conscientiousness in completing the study. Participants were then debriefed, had the headset removed, hair washed, and thanked for their time (see Fig. 4).

### Economic anxiety manipulation

Participants were asked to generate a headline for an ostensibly real news article on CBC.ca, the national public broadcaster Canada. In the economic anxiety condition, participants read an article that detailed a bleak and unsettling economic forecast for young Canadians. In the article, leading Canadian researchers conclude that a recession is imminent due to economic red flags such as wage stagnation, rising student debt, and declining long-term employment opportunities. Additionally, the researchers conclude that students would be hit hardest. As such, this article was tailored to cause economic anxiety in participants in our sample, i.e., young adult students. Participants in the No-Anxiety Control condition read an ostensibly real article from CBC.ca about a more neutral economic forecast that emphasized stability and a continuation of the status quo. Notably, both forecasts were based on real, publicly available economic predictions from financial news outlets. All participants were given three minutes to read the article and were later asked to submit their headline in a textbox before completing the BART.

### Balloon analogue risk task

Participants were told they would be entered into a lottery to win a $100 VISA gift card, and that they could increase their number of ballots (and chance of winning the $100 prize) by doing well in the computerized Balloon Analogue Risk Task (BART^30^). Participants pumped balloons across 20 trials, one balloon per trial, where each balloon had a variable and unknown pop-threshold. The average pop-threshold across trials was 15 pumps. Each pump slightly inflated the balloon and earned points but also brought the balloon closer to the pop-threshold. Popped balloons earned no points and participants could stop pumping at any time and collect the points earned on that trial. As in prior research using the BART, our primary measure of risk-taking was the average number of pumps on trials without explosions. We computed a second measure to tease apart strategic behavior and risk-taking—i.e., a coefficient of variability (COV). COV is computed here as the standard deviation across trials divided by average pumps across trials. Lower degrees of variability (i.e., lower COV scores) is thought to reflect more stringent adherence to a behavioral strategy and higher degrees of variability (i.e., higher COV scores) indicates more disinhibited decision-making. Consistent with the idea that COV indexes behavioral control, COV has been associated with greater executive function capacity in past research^57,58^. Finally, we computed a behavioral learning variable as total pumps on trials without explosions on the first five trials subtracted from total pumps on trials without explosions on the last five trials. We used the total score on non-explosion trials because the average score would not allow for people who learned to bank more points by avoiding explosion. For example, two participants could have the same average of 10 pumps, but one participant learned to avoid explosions and obtained 50 pumps in five trials, whereas the other participant did not learn to avoid explosions and obtained 20 pumps in two trials. Our variable thus captured this critical aspect of learning. Given that responding on the BART was generally risk-averse (i.e., almost all participants averaged below the optimal response of 15 pumps per trial), a higher score on this Learning score indicates that participants learned to approach optimal risk-taking.

### EEG recording and preprocessing

Continuous EEG was recorded using the 64 Ag–AgCl channel ActiCHamp EEG system (Brain Products), positioned according to the 10/10 system and digitized at a sampling rate of 512 Hz (24 bit precision; bandwidth: 0.1–100 Hz). During recording, signals were referenced to TP9 electrode positioned over the left mastoid. Offline, EEG was re-referenced to the average mastoids (TP9-TP10), down-sampled to 256 Hz, band-pass filtered between 0.1 and 30 Hz, and notch filtered at 60 Hz. To ensure we examined anxiety-specific activation, EEG was segmented into the last 60 s of the economic anxiety manipulation. Blinks were statistically removed using an automatic ocular correction algorithm^66^. Artifacts were then automatically detected using the following parameters: − 100 to + 100 μV min/max threshold, 50 μV maximum voltage step, 0.5 μV lowest allowed voltage (maximum–minimum) in 100 ms intervals. Contiguous artefact-free epochs of 2 s were extracted through a hamming window and overlapped by 75% to avoid data loss. Power spectra were calculated via fast Fourier transform and power values (in μV^2^) were averaged over all artefact-free and blink-free epochs. Absolute power was averaged for seven frequency bands^67^: delta (1.5–6 Hz), theta (6.5–8 Hz), alpha1 (8.5–10 Hz), alpha2 (10.5–12 Hz), beta1 (12.5–18 Hz), beta2 (18.5–21 Hz), and beta3 (21.5–30 Hz). Next, we segmented the continuous EEG recorded during the two 1-min eyes closed baseline measurement. We used the same preprocessing steps and calculated the absolute power values in the seven frequency bands. These data were used to control for baseline activation in our primary analyses.

Standardized low-resolution brain electromagnetic tomography (sLORETA^29^) was used to estimate the cortical sources of absolute power in each frequency band during the economic anxiety manipulation and during the eyes closed baseline recording. As opposed to dipole modelling, sLORETA computes activity as current density (A/m^2^) without assuming a predefined number of active sources. The sLORETA solution space consists of 6239 voxels (voxel size: 5 × 5 × 5 mm) restricted to cortex and hippocampi, as defined by the digitized Montreal Neurological Institute probability atlas. sLORETA has been reliably validated by research comparing sLORETA localization of EEG activity and functional Magnetic Resonance Imaging (fMRI^68,69^), Positron Emission Tomography data (PET^70^), and implanted electrodes in intracranial recordings^71^. Prior to analyses, sLORETA images were normalized to a total current density of one and log-transformed.

### Statistical analyses

We used sLORETA images of frequency-based EEG during the Economic Anxiety manipulation in two approaches to determine if anxiety-specific activation modulates risky-decision-making.

1. A whole brain approach, corrected for multiple comparisons: Whole-brain voxel-by-voxel independent groups *t*-tests of the sLORETA images were conducted separately for each EEG frequency band, comparing participants in the Economic Anxiety condition versus participants in the No-Anxiety Control condition, controlling for baseline eyes-closed sLORETA images. Correction for multiple testing for all 6239 voxels was implemented by means of a nonparametric randomization approach^72^. This approach estimates empirical probability distributions and the corresponding critical probability thresholds (corrected for multiple comparisons). We expected that the Economic Anxiety condition, compared to the No-Anxiety Control condition, would cause increased activation in regions associated with anxious worry, including the dmPFC/dACC, the vmPFC, and anterior insula. However, our whole brain approach allowed for examining other potential differences in intracerebral sources between conditions.
2. An a priori region of interest approach: In the whole brain analyses, activation in the anterior insula in the Economic Anxiety condition did not cross the critical probability threshold corrected for multiple comparisons, despite evidence of increased activation (*t*-values > 3). Given that the whole brain correction technique may be biased towards reducing Type I errors in the current study (e.g., correcting for a large number of comparisons in voxels not expected to be involved in either anxiety or decision-making, see^73^), and the anterior insula is reliably involved in both anxiety and risky decision making, we extracted sLORETA estimates of activation for each frequency band from the left and right anterior insula (MNI coordinates X = − [45] Y = 10 Z = 5). Recall that delta, theta and alpha1 bands are negatively related to cortical activation and alpha2 and beta 1–3 bands are positively related to cortical activation^32–34^. Consistent with this, preliminary analyses demonstrated that right anterior insula activation in alpha2, beta1, beta2, and beta3 frequency bands were all increased by the Economic Anxiety condition (all p’s < 0.05). To reduce comparisons, we averaged insula activation into Deactivation (delta, theta, alpha1) and Activation (alpha2, beta1, beta2, beta3) scores. We then conducted independent groups *t*-tests, Bonferroni corrected for 4 comparisons (right and left Insula, Activation, Deactivation), α = 0.0125.

We then conducted mediational analyses (conducted in SPSS 26 using the PROCESS macro^45^) to examine if anxiety-specific activation—i.e., activation caused by the Economic Anxiety manipulation—was associated with (1) average pumps adjusted for explosions (AvAdj), and (2) coefficient of variability for all trials (COV), given that this BART version involved a high number of explosions, and trials with explosions are equally relevant to a measure of behavioral control. Finally, we computed a BART learning variable from the first five and last five trials to examine if anxiety-specific activation impacted learning across the decision-making task.

## Supplementary Information

Supplementary Information.
