# Supplementary material for: Neural processes in antecedent anxiety modulate risk-taking behavior
Source: Sci Rep. 2021 Jan 29;11:2637. doi: 10.1038/s41598-021-82229-w (PMC7846834; doi:10.1038/s41598-021-82229-w)
Supplement: Supplementary file 1 — Supplementary Information. [file 41598_2021_82229_MOESM1_ESM.docx]

**Supplementary Material** **- *Neural Processes in Antecedent Anxiety Modulate Risk-Taking Behavior***

Kyle Nash, Josh Leota, & Alex Tran; Department of Psychology, University of Alberta

Edmonton, AB T6G 2R3, Canada

**S1.** Personality measures included: trait BIS and BAS (Carver & White, 1994), attachment style (Fraley et al., 2000), self-control (Tangney et al., 2004), the big five (Gosling et al., 2003), and aggression (Bryant & Smith, 2001).

**S2**. We wished to examine if performance on the intervening tasks between the economic anxiety manipulation and the BART did not impact the effect of the manipulation on behavior. The auditory oddball task was passive. As such, there are no performance variables. However, the second task was the color naming Stroop task. Notably, economic anxiety did not cause any change in performance in the Stroop task, as indexed by average reaction time (RT) to incongruent trials, average RT to congruent trials, RT standard deviation (SD) to incongruent trials, RT SD to congruent trials, accuracy to incongruent trials, accuracy to congruent trials, post-error slowing, and post-error accuracy rate (all *ps* > .05). Further, none of these variables mediated an indirect effect of condition on risk-taking or COV.

**References**

Bryant, F. B., & Smith, B. D. (2001). Refining the architecture of aggression: A measurement model for the Buss–Perry Aggression Questionnaire. *Journal of Research in Personality*, *35*(2), 138-167. https://doi.org/10.1006/jrpe.2000.2302

Carver, C. S., & White, T. L. (1994). Behavioral inhibition, behavioral activation, and affective responses to impending reward and punishment: the BIS/BAS scales. *Journal of Personality and Social Psychology*, *67*(2), 319.

Fraley, R. C., Waller, N. G., & Brennan, K. A. (2000). An item response theory analysis of self-report measures of adult attachment. *Journal of Personality and Social Psychology*, *78*(2), 350-365. https://doi.org/10.1037/0022-3514.78.2.350

Gosling, S. D., Rentfrow, P. J., & Swann Jr, W. B. (2003). A very brief measure of the Big-Five personality domains. *Journal of Research in Personality*, *37*(6), 504-528. https://doi.org/10.1016/S0092-6566(03)00046-1

Tangney, J. P., Baumeister, R. F., & Boone, A. L. (2004). High self‐control predicts good adjustment, less pathology, better grades, and interpersonal success. *Journal of Personality*, *72*(2), 271-324. https://doi.org/10.1111/j.0022-3506.2004.00263.x
